# Seven neurons memorizing sequences of alphabetical images via spike-timing dependent plasticity

**DOI:** 10.1038/srep14149

**Published:** 2015-09-16

**Authors:** Takayuki Osogami, Makoto Otsuka

**Affiliations:** 1 IBM, IBM Research - Tokyo, Tokyo, 103-8510, Japan

## Abstract

An artificial neural network, such as a Boltzmann machine, can be trained with the Hebb rule so that it stores static patterns and retrieves a particular pattern when an associated cue is presented to it. Such a network, however, cannot effectively deal with dynamic patterns in the manner of living creatures. Here, we design a dynamic Boltzmann machine (DyBM) and a learning rule that has some of the properties of spike-timing dependent plasticity (STDP), which has been postulated for biological neural networks. We train a DyBM consisting of only seven neurons in a way that it memorizes the sequence of the bitmap patterns in an alphabetical image “SCIENCE” and its reverse sequence and retrieves either sequence when a partial sequence is presented as a cue. The DyBM is to STDP as the Boltzmann machine is to the Hebb rule.

Artificial neural networks have been studied as means of automatic pattern recognition^1^,^2^,^3^ for a long time, and the recent breakthrough of deep learning^4^,^5^,^6^,^7^,^8^ has brought them once again to the forefront of artificial intelligence studies. A characteristic of an artificial neural network is associative memory, which stores multiple patterns in such a way that a particular pattern can be retrieved when it is given a cue such as a partial pattern^9^,^10^,^11^,^12^. The Hebb rule^13^ is used to train artificial neural networks, including Perceptrons^14^, Hopfield networks^10^, and Boltzmann machines^15^. In particular, it incrementally decreases the energy of the patterns to be stored in a Boltzmann machine, which in turn increases the likelihood that the Boltzmann machine generates those patterns^16^,^17^.

The Hebb rule used for artificial neural networks, however, has a fundamental shortcoming as a learning rule of biological neural networks, because the concept of time is largely missing from it. Specifically, it is independent of the precise timing of the spikes of neurons. A postulate that extends the Hebb rule is spike-timing dependent plasticity, or STDP^18^,^19^,^20^,^21^, which states that a synapse is strengthened if the spike of a pre-synaptic neuron precedes the spike of a post-synaptic neuron (*i.e.*, long term potentiation; LTP^22^,^23^), and the synapse is weakened if the temporal order is reversed (*i.e.*, long term depression; LTD). The existence of STDP was experimentally confirmed around the end of the last century^24^,^25^,^26^.

Here, we provide underpinnings for STDP as a learning mechanism by training an artificial neural network via STDP in such a way that the trained network exhibits associative memory for sequential patterns. Specifically, we model the dynamics of a biological neural network with an artificial neural network, which we refer to as a dynamic Boltzmann machine (DyBM). We train the DyBM by using an online learning rule that has some of the properties of STDP such as LTP and LTD. We sequentially present patterns to it and update its learnable parameters every time a pattern is presented. The DyBM then retrieves a particular sequence when associated cues are presented.

The structural features that distinguish a DyBM from a Boltzmann machine are conduction delays and memory units, which are illustrated in Fig. 1. A neuron is connected to another in a way that a spike from a pre-synaptic neuron, *i*, travels along an axon and reaches a post-synaptic neuron, *j*, via a synapse after a delay consisting of a constant period, *d*_*i,j*_. In the DyBM, a first-in first-out (FIFO) queue causes this conduction delay. The FIFO queue stores the values of the pre-synaptic neuron for the last *d*_*i,j*_ − 1 units of time. Each stored value is pushed one position toward the head of the queue when the time is incremented by one unit. The value of the pre-synaptic neuron is thus given to the post-synaptic neuron after the conduction delay. Moreover, the DyBM aggregates information about the spikes in the past into neural eligibility traces and synaptic eligibility traces^27^,^28^,^29^, which are stored in the memory units. The value of a neural eligibility trace increases when an associated neuron spikes and decreases otherwise. The value of a synaptic eligibility trace increases when the spike from a pre-synaptic neuron reaches a post-synaptic neuron and decreases otherwise. In the current study, we keep three eligibility traces with varying decay rates for each neuron and for each synapse. The use of varying decay rates is consistent with the approximation of the hyperbolic decay for long-term memory^30^,^31^,^32^.

Each neuron takes a binary value, 0 or 1, and the probability that a neuron takes the value 1 (*i.e.*, it spikes) at any moment depends on the previous values of the neurons as well as the values of the learnable parameters of the DyBM. Each neuron is associated with a learnable parameter called bias. The strength of the synapse between a pre-synaptic neuron and a post-synaptic neuron is represented by learnable parameters called weights. In order to represent LTP and LTD, the DyBM has an LTP weight and an LTD weight. We use an online gradient ascent method^33^,^34^ so as to maximize the likelihood of given sequential patterns. The learnable parameters are updated only on the basis of the information that is available at the associated synapse or neuron, and there is no need to store the whole sequence for learning via backpropagation through time^35^,^36^,^37^,^38^,^39^,^40^.

## Results

We trained a DyBM, consisting of seven neurons, in such a way that it could store multiple sequential patterns from alphabetical images. The DyBM would then retrieve a particular sequential pattern when part of it was presented as a cue, and it could also detect anomalies in a given sequence.

**Figure 2:**
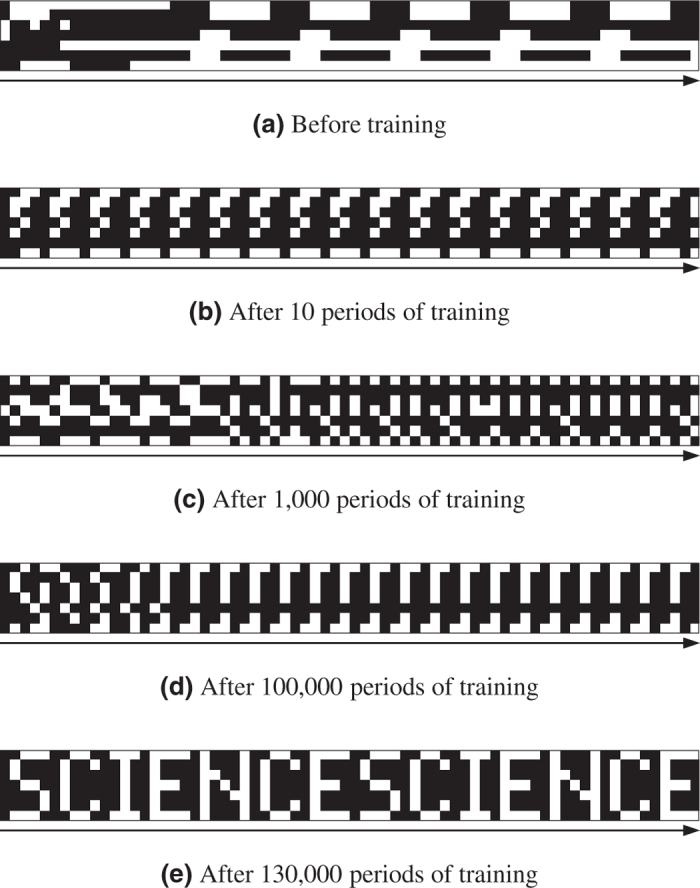
The DyBM learned the target sequence. (**a**) Before training began, the DyBM generated a sequence that was determined by the initial values of the learnable parameters. (**b**–**d**) In each period of training, we presented one period of the target sequence once to the DyBM. The DyBM gradually learned the target sequence as the training progresses from 10 periods to 100,000 periods. (**e**) After 130,000 periods, the DyBM generated the complete sequence.

First, we trained the DyBM with a single sequence of patterns from an alphabetical image, in this case “SCIENCE,” which is a 7-bit by 35-bit monochrome bitmap image. Each set of seven vertically aligned bits composed the pattern of the moment, and the sequence of 35 of these patterns composed the period (see Fig. 2e). We presented each pattern one at a time in sequence to the DyBM and updated its learnable parameters each time. We also updated the values of the eligibility traces and the FIFO queues each time a 7-bit pattern was presented. Each training period consisted of presenting one period of the sequence (*i.e.*, presenting “SCIENCE” once). We repeated the training period multiple times.

To show the progress of training in Fig. 2, we let the DyBM generate a sequence, for two periods of the target sequence, before and after the training as well as after some intermediate steps. Before training began (see Fig. 2a), the DyBM generated a sequence that had nothing to do with the target sequence, because the initial values of the learnable parameters were independent of the target sequence. Here, we let the DyBM generate a sequence in a deterministic manner. At each moment, a neuron spiked if and only if the probability that the neuron spikes was greater than 0.5. This corresponds to making the temperature (see Supplementary Table S1a) of the DyBM infinitesimally small. Although the values of the learnable parameters were fixed when the DyBM was generating a sequence, the eligibility traces and the FIFO queues were updated on the basis of the generated sequence. The DyBM thus generated varying patterns during the two periods.

After 10 to 100,000 periods of training (see Fig. 2b–d), the DyBM generated a sequence whose first five 7-bit patterns composed part of “S.” Here, we let the DyBM start generating a sequence without refreshing the eligibility traces or the FIFO queues that were updated during the training. The sequence presented during the training thus served as a cue for generating the sequence shown in Fig. 2. Training the DyBM completed in 85 seconds after 130,000 periods (see Fig. 2e), at which point the DyBM generated the complete sequence of the target pattern.

**Figure 3:**
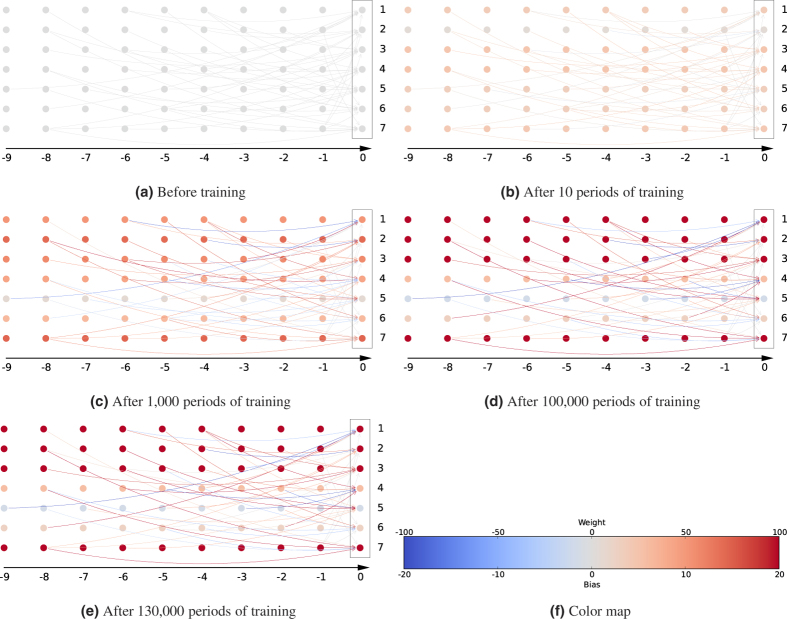
The DyBM updated the values of its learnable parameters during training with “SCIENCE.” The color of a circle in a box shows the bias of a neuron. The color of an arrow shows the LTP weight (*u*_*i,j*,1_ + *u*_*i,j*,2_ + *u*_*i,j*,3_ with the notations in Supplementary Table S1b) from a pre-synaptic neuron, *i*, to a post-synaptic neuron, *j*. The conduction delay from neuron *i* to neuron *j* is denoted by *d*_*i,j*_, and an arrow is drawn from the *i*-th circle in the column labeled −*d*_*i,j*_ to the *j*-th circle in the box. Here, the conduction delay is sampled independently from the uniform integer distribution with support [1, 9] (see Supplementary Table S2). (**a**) The initial values of the learnable parameters were sampled independently from the normal distribution with mean 0.0 and standard deviation 0.1. (**b**–**e**) Some of the learnable parameters were strengthened while others were weakened as the training progresses from 10 periods to 130,000 periods. The color map in (**f**) denotes the values of the learnable parameters.

Figure 3 illustrates how the learnable parameters of the DyBM were updated during the training. The seven vertically aligned circles in a box represent the seven neurons, which are arranged from the top to the bottom in the order corresponding to the 7-bit patterns. The color of the circles represents the value of the associated bias in accordance with the color map shown in Fig. 3f. The neurons corresponding to the red circles are more likely to spike than others if the other conditions are equivalent. The arrows in Fig. 3 represent the LTP weights. As the conduction delay from pre-synaptic neuron *i* to post-synaptic neuron *j* is denoted by *d*_*i,j*_, we draw an arrow from the *i*-th circle in the column labeled −*d*_*i,j*_ to the *j*-th circle in the box. The conduction delay was sampled independently from an integer uniform distribution between 1 and 9 (see Supplementary Table S2). The color of an arrow represents the value of the LTP weight in accordance with the color map in Fig. 3f. A post-synaptic neuron, *j*, that is connected with a red arrow from a pre-synaptic neuron, *i*, is more likely to spike than others, if the other conditions are equivalent, shortly after the spike from the pre-synaptic neuron reaches the post-synaptic neuron after the conduction delay *d*_*i,j*_.

Before training began (see Fig. 3a), the learnable parameters were independent of the target sequence and sampled independently from the normal distribution with mean 0.0 and standard deviation 0.1. The DyBM gradually learned appropriate values of the bias and the LTP weight as the training progresses (see Fig. 3b–e). It also learned the LTD weight (see Supplementary Fig. S3).

Next, we show that the trained DyBM can detect anomalies^41^ in a sequence. We presented the sequence “SCIENSESCIENCE” to the DyBM that was trained to generate the sequence shown in Fig. 2e. This presented sequence has an anomaly in that the second “C” of the first instance of “SCIENCE” is replaced with “S.” We did not train the DyBM but did update the eligibility traces and FIFO queues, while showing it the anomalous sequence.

**Figure 4:**
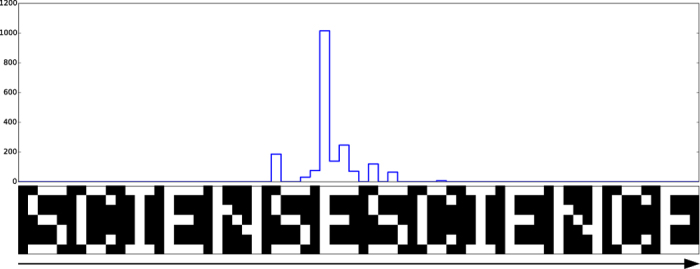
The DyBM trained with “SCIENCE” detected an anomaly in “SCIENSESCIENCE.” After training the DyBM for 130,000 periods, we presented the DyBM with the anomalous sequence shown at the bottom of the figure. The top part shows the negative log-likelihood, predicted by the DyBM, for each of the seven-bit patterns in the anomalous sequence.

Figure 4 shows that the DyBM detected the anomaly in the sequence. The lower part of the figure shows the anomalous sequence. The plot in the upper part of Fig. 4 shows the negative log-likelihood (score of anomaly), predicted by the DyBM, for each of the 7-bit patterns in the anomalous sequence. The higher the negative log-likelihood of a 7-bit pattern is, the less likely it is that the DyBM generates that 7-bit pattern, given the preceding patterns. The predicted negative log-likelihood increased by orders of magnitude when the DyBM was presented with the first 7-bit pattern of the anomalous “S.” This 7-bit pattern itself is not an anomaly and appears elsewhere with a small negative log-likelihood. The negative log-likelihood was indeed predicted to be small when the anomalous “S” is replaced with the normal “C.” The initial patterns of the second “SCIEN” were predicted to have a high negative log-likelihood because they anomalously follow “SE.”

Finally, we show that the DyBM can store multiple sequences and retrieve a particular sequence when an associated cue is presented to it. Here, we used two sequences, forward and reverse, from the alphabetical image “SCIENCE.” The forward sequence was “SCIENCE,” as in the previous experiments. The reverse sequence was created by reversing the order of the 7-bit patterns in the forward sequence, and thus is a sequential pattern from the mirror image of “SCIENCE.” We trained the DyBM by presenting it with one of the two sequences in each iteration until it correctly retrieved that sequence when a partial sequence was presented as a cue. The cue for the forward sequence (forward cue) was the sequential pattern “SCIEN.” The cue for the reverse sequence (reverse cue) was the sequential pattern of the mirror image of “IENCE.”

**Figure 5:**
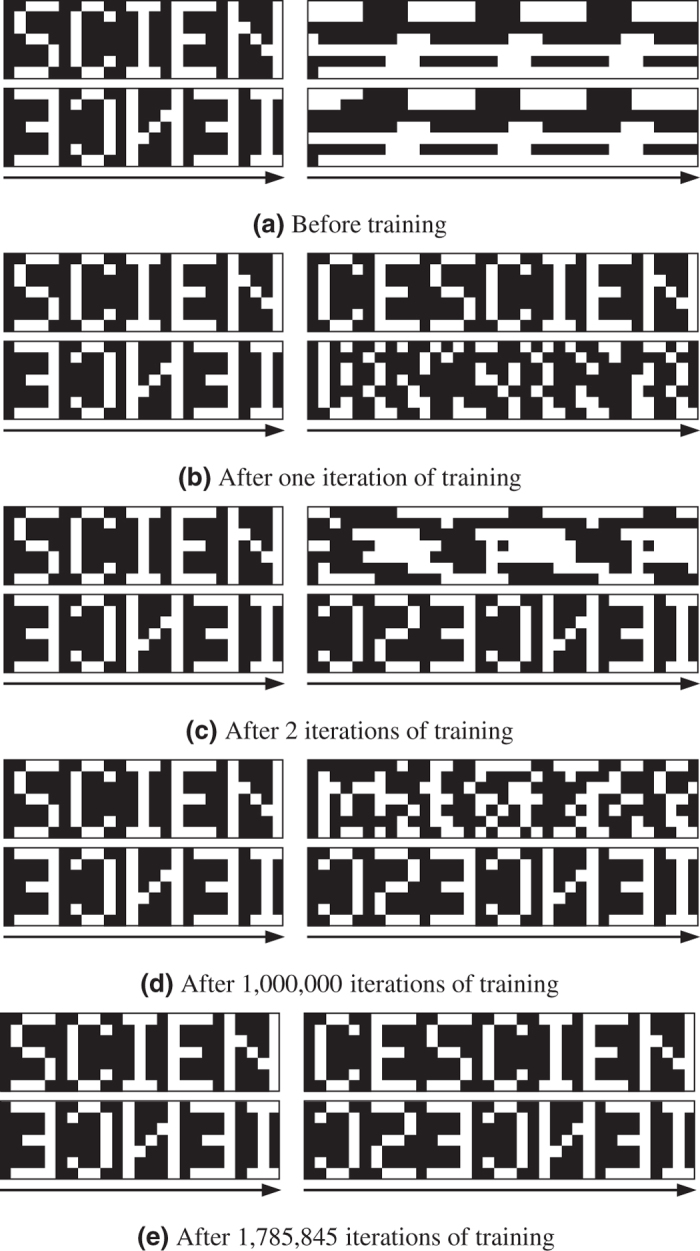
The DyBM learned two target sequences and retrieved a particular sequence when an associated cue was presented. The DyBM generated the right sequence after the left sequence was presented as a cue. (**a**) The DyBM did not generate the target sequences prior to training. (**b**) The DyBM learned the first target sequence “SCIENCE” in the first iteration and retrieved it when its partial sequence was presented as a cue. (**c**) After the second iteration, the DyBM retrieved the second target sequence of the mirror image of “SCIENCE” but not the first. (**d**) After one million iterations, the DyBM learned the second target sequence, while keeping a partial memory of the first. (**e**) The DyBM eventually retrieved one of the two target sequences, depending on the cue.

Figure 5 shows how the DyBM learned the target sequences as the training progresses. The left images are the cues presented to the DyBM, and the right images are the sequential patterns that the DyBM generated after the corresponding cues were presented. We did not train the DyBM but did update its eligibility traces and FIFO queues when it was presented with cues or when it was generating sequential patterns. Here, the values of the eligibility traces and the FIFO queues were reset to zero before a cue was presented. This corresponds to presenting a sequence of zeros or a sequential pattern of a blank image to the DyBM for a sufficiently long period. Before training began (Fig. 5a), the DyBM generated neither of the target sequences, because the initial values of its learnable parameters were independent of the target sequences.

In the first iteration of training, we kept presenting the forward sequence to the DyBM. After the first iteration, the DyBM generated the forward sequence when the forward cue was presented (the upper part of Fig. 5b). At this point, the DyBM has not learned the reverse sequence. When the DyBM was presented with the reverse cue, it generated a sequence that starts with a part of “E” (the lower part of Fig. 5b). This happened because “E” follows “I” in the forward sequence, and the reverse cue ends with the mirror image of “I,” which has vertical symmetry.

In the second iteration, we kept presenting the reverse sequence to the DyBM. After the second iteration, the DyBM generated the reverse sequence when the reverse cue was presented (the lower part of Fig. 5c). However, this DyBM lost its memory about the forward sequence and did not retrieve it when the forward cue was presented (the upper part of Fig. 5c). After the second iteration, we presented the forward sequence to the DyBM in odd iterations and the reverse sequence in even iterations. Figure 5d shows the sequences generated by the DyBM after one million iterations. The DyBM generated the reverse sequence, which was presented in the last iteration of training, but not the forward sequence.

**Figure 6:**
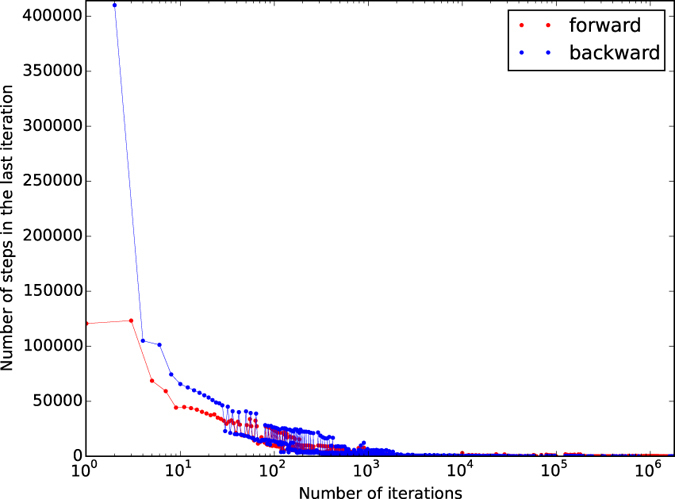
The DyBM quickly learned or recalled a target sequence that it had forgotten in previous iterations. The figure plots the number of periods that the DyBM took to learn a target sequence in each iteration of training in Fig. 5 against the number of iterations that have been completed.

Training the DyBM completed in 223 minutes after 1,785,845 iterations, at which point the DyBM learned the forward sequence without losing its memory of the reverse sequence learned in the preceding iteration. Figure 5e shows that the completely trained DyBM correctly generated the target sequences in accordance with the cues. Figure 6 shows the number of periods needed in each iteration of the training in Fig. 5. The first iteration finished in 120,701 periods. Each iteration took at most 5,837 periods after 1,000 iterations and at most 233 periods after 1,000,000 iterations. The DyBM learned a sequence quickly if it had already learned that sequence and forgotten it.

## Additional results

The DyBM is limited neither to seven neurons nor to alphabetical images. Here, we apply the DyBM to additional two sequences. We use varying number of neurons, depending on the dimension of the bit-patterns. First, we trained the DyBM with the sequence that represents the motion picture of human evolution, illustrated in Supplementary Fig. S1f. Here, we used 20 neurons in the DyBM to learn this sequence of 20-bit patterns having the period of 41 units of time. Training completed in 19 seconds. Supplementary Fig. S1 shows the sequences that were generated by the trained DyBM. Second, we trained the DyBM with the sequence that represents a music, a simplified version of *Ich bin ein Musikante*, illustrated in Supplementary Fig. S2f. Here, we used 12 neurons in the DyBM, corresponding to 12 distinct notes. Training completed in 28 minutes. Supplementary Fig. S2 shows the sequences that were generated by the trained DyBM.

## Discussion

The DyBM is trained by incrementally increasing the likelihood of the given dynamic patterns according to a learning rule that exhibits some of the properties of STDP. This training is analogous to training a conventional Boltzmann machine according to the Hebb rule such that the likelihood of the given static patterns is incrementally increased. Unlike the learning rules for the existing models that extend Boltzmann machines to deal with sequential patterns^32^,^42^,^43^,^44^,^45^, our learning rule exactly increases the likelihood without approximations. The recent success of artificial neural networks in a number of engineering applications suggests that exact learning will be very useful^46^,^47^. The sequence of 7-bit patterns of the alphabetical image “SCIENCE” has a period of 35, which is significantly longer than the conduction delay, which ranges between 1 and 9. Learning this sequence involves finding a complex interaction among the neurons, FIFO queues, and eligibility traces by adjusting the values of the learnable parameters. A slight deviation in the learnable parameters from the appropriate values results in a failure to generate the target sequences, as is evident in Fig. 2 and Fig. 3.

Each learnable parameter of a DyBM can be updated only with information that is locally available in space and time, which is an important aspect of a learning rule of biological neurons^48^ and is also a desirable property when it comes to implementation as hardware. Specifically, the bias of a neuron is updated with the eligibility traces and FIFO queues associated with that neuron, its pre-synaptic neuron, or its post-synaptic neurons. The weight of a synapse is also updated with the information associated with its pre-synaptic neuron and its post-synaptic neuron. Although the DyBM learns the conditional probability distribution of the current patterns given the preceding patterns, it does not store all of the preceding patterns. Instead, the information about the preceding patterns is aggregated into the latest values of the eligibility traces and FIFO queues. The eligibility traces, either neural or synaptic, and the FIFO queues are also updated only with local information, *i.e.*, whether or not a spike is generated or has arrived at an associated neuron.

Similar to standard recurrent neural networks^40^,^49^,^50^, the DyBM can be unfolded though time^51^,^52^. The unfolded DyBM is a Boltzmann machine having an infinite number of units, each representing the value of a neuron at a particular time. In particular, the units representing the most recent values of neurons are not connected to each other. The weights on connections share a finite number of values and are trained via discriminative learning^53^.

To date, STDP had limited success in engineering applications primarily because of the lack of sufficient underpinnings. This situation is analogous to the history of the Hebb rule. Although the Hebb rule motivated early studies on artificial neural networks prior to the 1980’s^14^, it had limited success until it was given an underpinning in the form of the Hopfield network^10^ and the Boltzmann machine^15^,^16^,^17^. The DyBM does the same for STDP. In fact, the learning rule of the DyBM also exhibits a form of homeostatic plasticity for keeping the spiking probability relatively constant^54^,^55^. The DyBM thus provides an underpinning for homeostatic STDP.

## Methods

Here, we describe the details of the dynamic Boltzmann machine (DyBM), the learning rule for the DyBM, and the parameters of the DyBM used in the experiments.

### Dynamic Boltzmann machine

A DyBM consists of a set of neurons having memory units and first-in-first-out (FIFO) queues. Let *N* be the number of neurons. Each neuron takes a binary value of either 0 or 1 at each moment. For 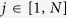, let 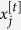 be the value of the *j*-th neuron at time *t*.

A neuron, 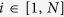, may be connected to another neuron, 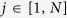, with a FIFO queue of length *d*_*i,j*_ − 1, where *d*_*i,j*_ is the axonal or synaptic delay of conductance, or conduction delay, from pre-synaptic neuron *i* to post-synaptic neuron *j*. Note that any neuron can be called a pre-synaptic neuron and a post-synaptic neuron, depending on the synapse under consideration. We assume *d*_*i,j*_ ≥ 1. At each moment *t*, the tail of the FIFO queue holds 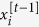, and the head of the FIFO queue holds 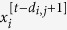. As the time progresses by one unit, the value at the head of the FIFO queue is removed, the remaining values in the FIFO queues are pushed toward the head by one position, and a new value is inserted at the tail of the FIFO queue. We allow a neuron to be connected to itself via a FIFO queue.

Each neuron stores a fixed number, *L*, of neural eligibility traces. For 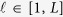 and 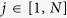, let 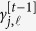 be the 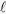-th neural eligibility trace of the *j*-th neuron immediately before time *t*:


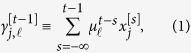


where 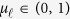 is the decay rate for the 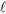-th neural eligibility trace. That is, the neural eligibility trace is the weighted sum of the past values of that neuron, where the recent values have greater weights than older ones.

Each neuron also stores synaptic eligibility traces, where the number of the synaptic eligibility traces depends on the number of the neurons that are connected to that neuron. Namely, for each of the (pre-synaptic) neurons that are connected to a (post-synaptic) neuron *j*, the neuron *j* stores a fixed number, *K*, of synaptic eligibility traces. For 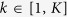, let 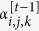 be the *k*-th synaptic eligibility trace of neuron *j* for pre-synaptic neuron *i* immediately before time *t*:


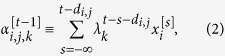


where 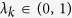 is the decay rate for the *k*-th synaptic eligibility traces. That is, the synaptic eligibility trace is the weighted sum of the values that has reached that neuron, *j*, from a pre-synaptic neuron, *i*, after the conduction delay, *d*_*i,j*_. Again, the recent values have greater weights than older ones.

The values of the eligibility traces stored at neuron *j* are updated locally at time *t* using the value of neuron *j* at time *t* and the values that have reached neuron *j* at time *t* from its pre-synaptic neurons. Specifically,


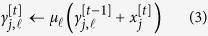



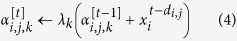


for 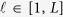 and 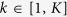, and for neurons *i* that are connected to *j*.

The DyBM has learnable parameters that are updated during training, in addition to the structural parameters (Supplementary Table S1a) and variables (Supplementary Table S1c), which have been introduced in the preceding. The learnable parameters of the DyBM are the bias and weight (Supplementary Table S1b). Specifically, each neuron, *j*, is associated with a bias, *b*_*j*_. Each synapse, or each pair of neurons that are connected via a FIFO queue, is associated with the weight of long term potentiation (LTP weight) and the weight of long term depression (LTD weight). The LTP weight from a (pre-synaptic) neuron, *i*, to a (post-synaptic) neuron, *j*, is characterized with *K* parameters, *u*_*i,j,k*_ for 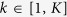. The *k*-th LTP weight corresponds to the *k*-th synaptic eligibility trace for 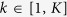. The LTD weight from a (pre-synaptic) neuron, *i*, to a (post-synaptic) neuron, *j*, is characterized with *L* parameters, 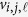 for 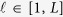. The 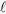-th LTD weight corresponds to the 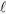-th neural eligibility trace for 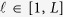. The learnable parameters are collectively denoted as *θ*.

We now define the energy of the DyBM, using the notations introduced in the preceding. Similar to the conventional Boltzmann machine^15^,^16^,^17^, the energy of the DyBM determines what patterns of values that the DyBM is more likely to generate. Contrary to the conventional Boltzmann machine, the energy associated with a pattern at a moment depends on the patterns that the DyBM has previously generated. Let 
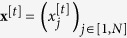
 be the vector of the values of the neurons at time *t*. Let 
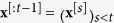
 be the sequence of the values of the DyBM before time *t*. The energy of the DyBM at time *t* depends not only on **x**^[*t*]^, but also on **x**^[:*t*−1]^, which is stored as eligibility traces in the DyBM. Let 
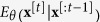
 be the energy of the DyBM at time *t*. The lower the energy of the DyBM with particular values **x**^[*t*]^, the more likely the DyBM takes those values.

The energy of the DyBM can be decomposed into the energies of the individual neurons at time *t*:


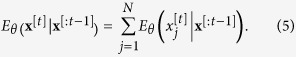


The energy of neuron *j* at time *t* depends on the value it takes as follows:


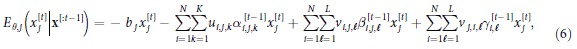


where we define


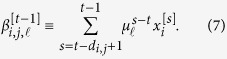


The first term of the right side of equation (6) shows that, roughly speaking, a neuron having a large positive bias is likely to spike 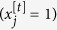 at any time *t*, because its energy tends to be low when it spikes. More precisely, the energy of the neuron is determined by the balance among the four terms on the right side of equation (6).

The second term of the right side corresponds to LTP. Consider a pair of a pre-synaptic neuron, *i*, and a post-synaptic neuron, *j*, whose LTP weight, *u*_*i,j,k*_ for 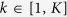, has a large positive value. Then *j* is likely to spike at time *t*, if the spikes from *i* have arrived shortly before time *t*, which makes 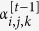 large for 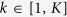.

The third term of the right side corresponds to LTD. LTD suggests that a post-synaptic neuron, *j*, is unlikely to spike shortly before a spike from a pre-synaptic neuron, *i*, reaches *j*. The corresponding LTD weight, 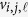 for 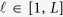, controls the strength of LTD for that synapse. The FIFO queue holds the spikes generated by *i* for the last *d*_*i,j*_ − 1 units of time, and those spikes reach *j* within a short period of at most *d*_*i,j*_ − 1 units of time. Here, 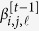, as defined in equation (7), takes a positive value when the FIFO queue from neuron *i* to neuron *j* has spikes (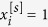 for some of 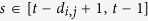). The more spikes the FIFO queue has, and the closer to the head of the FIFO queue those spikes are, the larger the value of 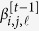 will be. Equation (6) suggests that the post-synaptic neuron, *j*, is unlikely to spike, when 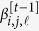 is large and the corresponding LTD weight, 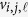 for 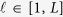, has a large positive value.

The last term of the right side can also be considered LTD. The intuition is that the third term only considers the spikes that are going to reach the post-synaptic neuron within the period of conduction delay, while the last term considers the spikes that are going to arrive after the conduction delay. In the last term, neuron *i* plays the role of a post-synaptic neuron, and neuron *j* is a pre-synaptic neuron, contrary to their roles in the third term. The LTD weight is thus 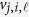, rather than 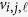, for 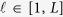.

Consider a synapse from a pre-synaptic neuron, *j*, to a post-synaptic neuron, *i*, that has a large positive value of 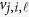 for 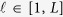. The neural eligibility trace, 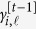, stores information about the spikes that the post-synaptic neuron *i* generated before time *t*. Namely, the value of 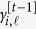 is high when neuron *i* spikes shortly before time *t*. The post-synaptic neuron is unlikely to have spiked shortly before time *t*, if the pre-synaptic neuron spikes at time *t*, which will reach the post-synaptic neuron after the conduction delay. Specifically, if *i* spiked at time *t* − *δ* and *j* spiked at time *t*, then that spike from *j* would reach *i* after the conduction delay, at time *t* + *d*_*j,i*_, which is *δ* + *d*_*j,i*_ units of time after *i* spiked. LTD suggests that such a pair of spikes is unlikely to be generated when *δ* + *d*_*j,i*_ is small. The causality is, however, that the pre-synaptic neuron is unlikely to spike at time *t* (*i.e.*, 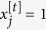) if the post-synaptic neuron has recently spiked.

The probability that a neuron, *j*, takes a particular value, 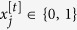, at time *t* depends on **x**^[:*t*−1]^, through the values of the eligibility traces, and can be expressed as follows:


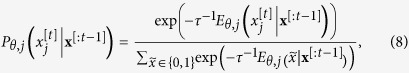


where *τ* is a parameter called temperature. The values of the neurons at time *t* are conditionally independent of each other, given **x**^[:*t*−1]^. Then the probability that the DyBM takes **x**^[*t*]^ at time *t* can be expressed as follows:
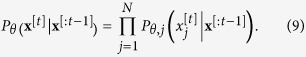


The corresponding log-likelihood is given by


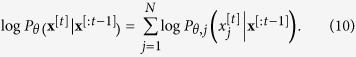


We generated the sequential patterns in Fig. 2 and Fig. 5 by using equation (8) with an infinitesimally small temperature. Specifically, we let *τ* → 0, so that equation (8) only takes 0 or 1. At each moment *t*, the values of the neurons, 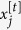 for 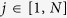, are deterministically generated. Those values are used to update the eligibility traces, before the values at the next moment, *t* + 1, are generated.

Figure 4 shows the magnitudes of the log-likelihood (or negative log-likelihood) calculated using equation (10). The values, 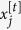 for 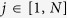, presented to the DyBM to calculate its log-likelihood at time *t* are used to update the eligibility traces, before the values at the next moment, *t* + 1, are presented.

### Learning rule

The probability distribution of the values that the DyBM generates depends on the values of the learnable parameters, *θ*, as is evident in equation (8). Below, we describe how to train those learnable parameters.

When the DyBM is presented with the values, **x**^[*t*]^, at time *t*, we update its learnable parameters in the direction of increasing log-likelihood:


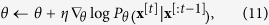


where *η* is the learning rate. This in turn increases the log likelihood of the sequence of the values up to time *t*:


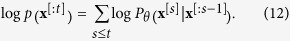


During training, we keep the temperature at *τ* = 1.

Specifically, when the value, 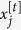, is presented to a neuron, *j*, its bias is updated as follows:


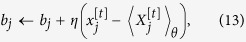


where


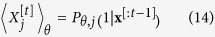


denotes the expectation of the value that *j* generates at time *t* given the values of the parameters and variables of the DyBM immediately before that time. Namely, the bias is increased if the value presented to the neuron is greater than what is expected. Otherwise, the bias is decreased. The magnitude of the update is proportional to the difference between the presented value and the expected value. The learning rule for the bias is similar to the Hebb rule^16^,^17^ except that, here, the expression of expectation (14) depends on the past values of the neurons via equation (8). The term with the expectation has the role of homeostatic plasticity, which keeps the spiking probability relatively constant^54^,^55^. Analogous terms of homeostatic plasticity will appear in the following learning rule for the other parameters.

The LTP weight is increased or decreased analogously to a bias as follows:


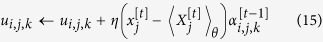


for each 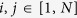 and 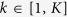. Now, the magnitude of the update is also proportional to the corresponding synaptic eligibility trace, 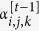. The learning rule for the LTP weight takes a form that is analogous to the REINFORCE algorithm^56^, where *η* is called a learning rate factor, 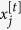 corresponds to reinforcement, 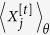 corresponds to the reinforcement baseline, and 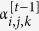 is called a characteristic eligibility, although the particular forms of individual factors are unique to the DyBM.

We can also express equation (15) as follows:


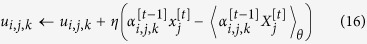


Namely, the LTP weight is increased if the product of the presented value and the value of the synaptic eligibility trace is greater than what is expected. Otherwise, the LTP weight is decreased. Increasing *u*_*i,j,k*_ results in increasing the probability that neuron *j* spikes particularly at the time *s* when 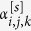 is high. This is what we expect with LTP.

The LTD weight is increased or decreased, depending on two terms:


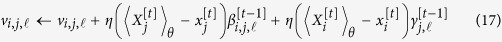


for each 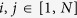, and 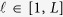. Again, we can express equation (17) equivalently as follows:


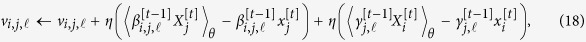


which can then be divided into two steps:


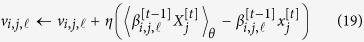



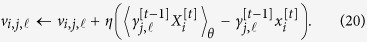


Equation (19) involves the product of 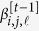 and 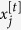, where we should recall that the value of 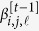 in equation (7) depends on the spikes traveling from neuron *i* to neuron *j*. The LTD weight is increased if the expected value of this product is greater than the corresponding observed value. Increasing *v*_*i,j,l*_ decreases the probability that neuron *j* spikes at time *t* when 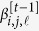 is high, which is what we expect with LTD. Equation (20) involves the product of 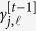, the neural eligibility trace of neuron *j*, and 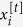, the value of neuron *i*. The LTD weight is increased if the expected value of this product is greater than the corresponding observed value. Increasing *v*_*i,j,l*_ decreases the probability that neuron *i* spikes at time *s* when 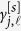 is high.

We trained the DyBM with the online gradient ascent method^33^ before letting it generate the sequential patterns shown in Fig. 2 and Fig. 5 and before presenting it with the anomalous sequence shown in Fig. 4. Specifically, after presenting the value, **x**^[*t*]^, to the DyBM at time *t*, we update its bias according to equation (13), its LTP weight according to equation (15), and its LTD weight according to equations (19 and 20). Before presenting the next value, **x**^[*t*+1]^, to the DyBM, we also update the eligibility traces according to equations (1 and 2) as well as the FIFO queues.

We initially set the learning rate at *η* = 1 and adjust it for each parameter as the training progresses, using AdaGrad^34^. Specifically, for *m* ≥ 0, let *η*_*m*_ be the learning rate of a parameter, *ξ* ∈ *θ*, after *ξ* is updated *m* times. We update *ξ* according to


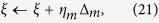


where Δ_*m*_ is the corresponding derivative of the log-likelihood, equation (10), with respect to *ξ*. For *m* ≥ 1, we adjust *η*_*m*_ according to


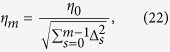


where we set *η*_0_ = 1. More precisely, the learning rates for the LTD weight are adjusted independently between equation (19) and equation (20) for each 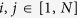 and 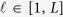.

### Parameters

Throughout the experiments, the *N* neurons were densely connected to each other, and the conduction delay was sampled from an integer uniform distribution between 1 and 9 (see Supplementary Table S2). In particular, each neuron was connected to itself via a FIFO queue. Each neuron held *L* = 3 neural eligibility traces, whose decay rates were *μ*_1_ = 0.25, *μ*_2_ = 0.5, or *μ*_3_ = 0.75. A neuron also held *K* = 3 synaptic eligibility traces, whose decay rates were *λ*_1_ = 0.25, *λ*_2_ = 0.5, or *λ*_3_ = 0.75, for each of the *N* FIFO queues coming into that neuron. The values of these structural parameters were fixed.

Before training, we set the initial values of the variables (Supplementary Table S1c) to 0. This corresponds to presenting a sequence of blank patterns, or zero vectors, for a sufficiently long period before the training. We sample the initial values of the learnable parameters (Supplementary Table S1b) independently from a normal distribution with mean 0.0 and standard deviation 0.1.

We implemented the algorithm for training DyBMs in Java^™^ and executed it on a Java Virtual Machine with the default setting for the maximum heap size (2 GB) and ran it on a single thread of an Intel Xeon E5-2670 processor on a workstation equipped with Microsoft Windows 7 Professional edition.

## Additional Information

**How to cite this article**: Osogami, T. and Otsuka, M. Seven neurons memorizing sequences of alphabetical images via spike-timing dependent plasticity. *Sci. Rep.* **5**, 14149; doi: 10.1038/srep14149 (2015).

## Supplementary Material

Supplementary Information

## Figures and Tables

**Figure 1:**
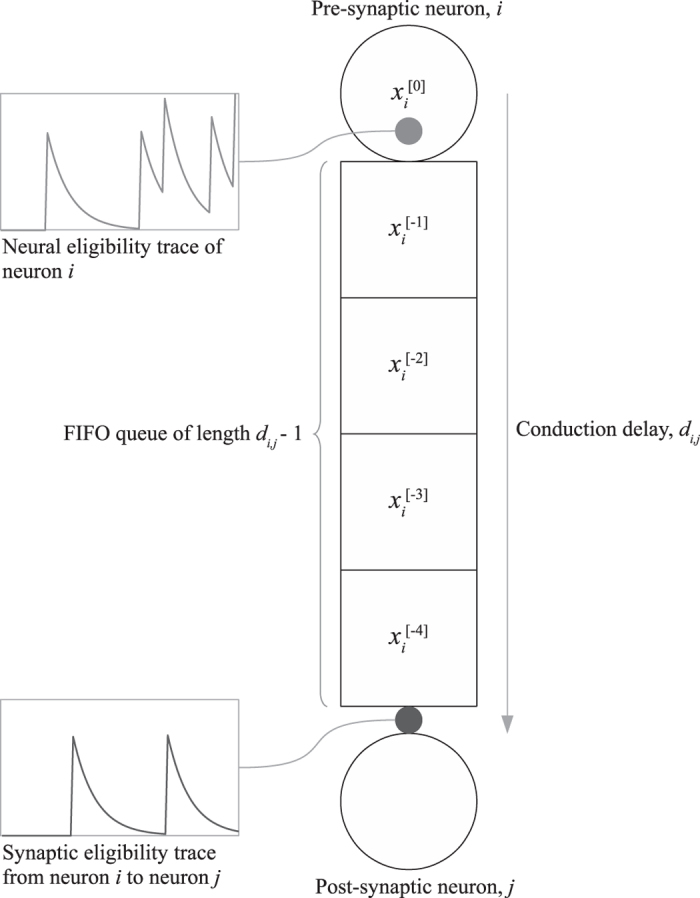
A DyBM consists of a network of neurons and memory units. A pre-synaptic neuron is connected to a post-synaptic neuron via a FIFO queue. The spike from the pre-synaptic neuron reaches the post-synaptic neuron after a constant conduction delay. Each neuron has the memory unit for storing neural eligibility traces, which summarize the neuron’s activities in the past. A synaptic eligibility trace is associated with a synapse between a pre-synaptic neuron and a post-synaptic neuron, and summarizes the spikes that have arrived at the synapse, via the FIFO queue, from the pre-synaptic neuron.
