# Supplementary material for: Seven neurons memorizing sequences of alphabetical images via spike-timing dependent plasticity
Source: Sci Rep. 2015 Sep 16;5:14149. doi: 10.1038/srep14149 (PMC4570975; doi:10.1038/srep14149)
Supplement: Supplementary Information [file srep14149-s1.pdf]

Supplementary information to:
Seven neurons memorizing sequences of alphabetical images via spike-timing dependent plasticity

Takayuki Osogami[1,*,+] **and Makoto Otsuka**[1,+]

[1]IBM, IBM Research - Tokyo, Tokyo, 135-8511, Japan
[*]osogami@jp.ibm.com
[+]these authors contributed equally to this work

Here we provide three supplementary figures and two supplementary tables for “Seven neurons memorizing sequences of alphabetical images via spike-timing dependent plasticity.”

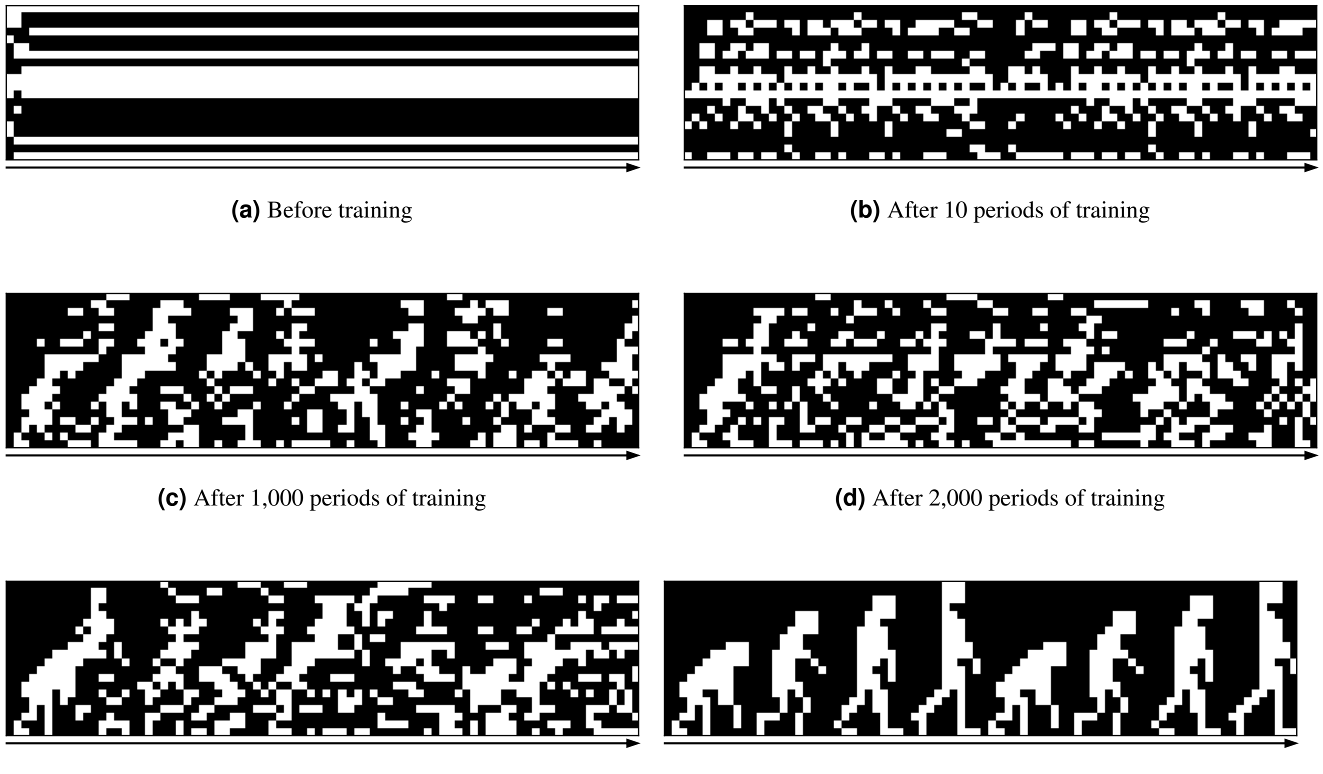

(a) Before training

(b) After 10 periods of training

(c) After 1,000 periods of training

(d) After 2,000 periods of training

(e) After 4,000 periods of training

(f) After 5,000 periods of training

Fig. S1. The DyBM learned the target sequence of human evolution. (a) Before training began, the DyBM generated the sequential pattern that was determined by the initial values of the parameters. (b-e) In each period of training, we presented one period of the target sequence once to the DyBM. The DyBM gradually learned the target sequence, as the training progresses from 10 periods to 4,000 periods. (f) After 5,000 periods, the DyBM generated the complete sequence of human evolution.

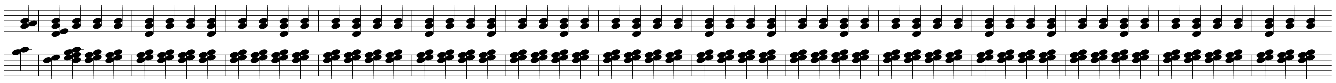

(a) Before training

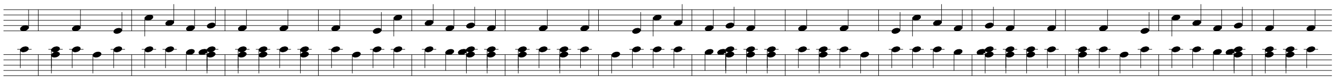

(b) After 10 periods of training

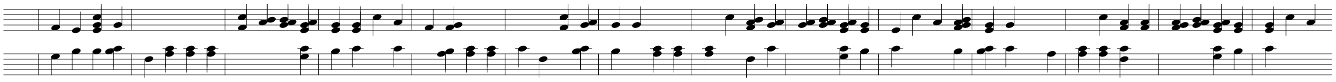

(c) After 1,000 periods of training

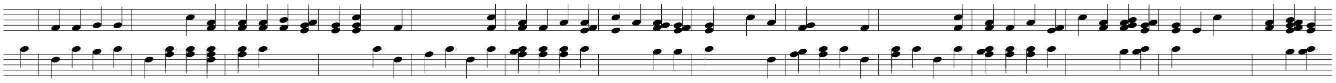

(d) After 10,000 periods of training

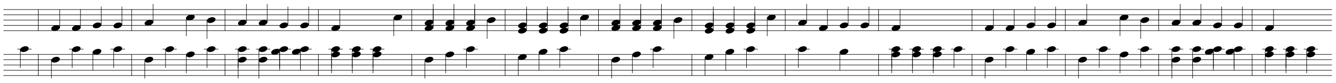

(e) After 100,000 periods of training

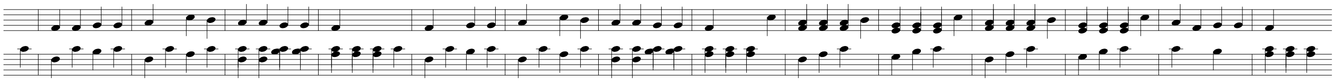

(f) After 900,000 periods of training

Fig. S2. The DyBM learned the target music. (a) Before training began, the DyBM generated the music that was determined by the initial values of the parameters. (b-e) In each period of training, we presented one period of the target music once to the DyBM. The DyBM gradually learned the target music, as the training progressed from 10 periods to 100,000 periods. (f) After 900,000 periods, the DyBM generated the target music.

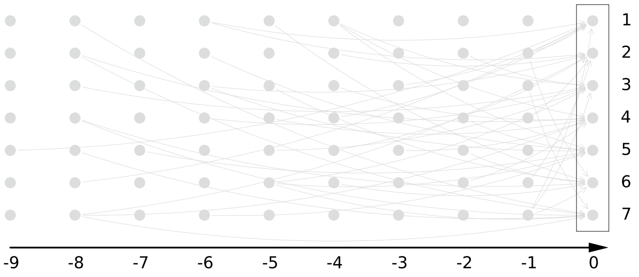

(a) Before training

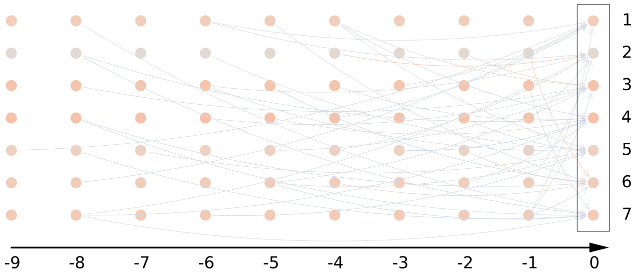

(b) After 10 periods of training

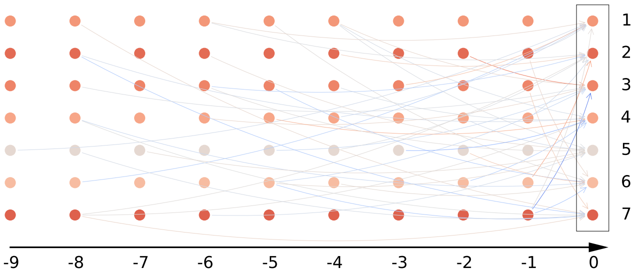

(c) After 1,000 periods of training

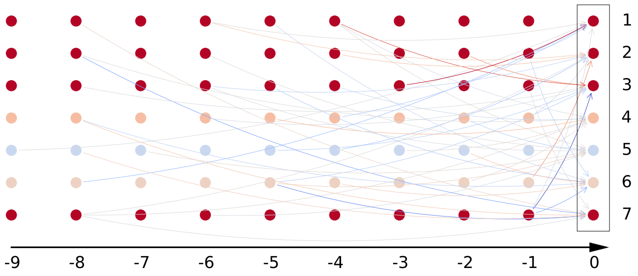

(d) After 100,000 periods of training

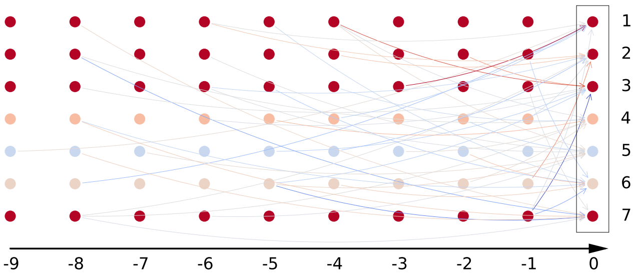

(e) After 130,000 periods of training

Fig. S3. The figure shows LTD weight, analogously to LTP weight in Fig. 3. Here, the color of an arrow shows the LTD weight ($v_{i,j,1} + v_{i,j,2} + v_{i,j,3}$ with the notations in Supplementary Table S1b) from a pre-synaptic neuron, i, to a post-synaptic neuron, j.

Notation	Definition
N	The number of neurons
L	The number of neural eligibility traces for each neuron
K	The number of synaptic eligibility traces for each pair of neurons
$d_{i,j}$	The delay from neuron i to neuron j
μ_ℓ	The decay rate of the ℓ-th neural eligibility trace ($\ell \in [1, L]$)
λ_k	The decay rate of the k-th synaptic eligibility trace ($k \in [1, K]$)
τ	Temperature

(a) Structural parameters

Notation	Definition
b_j	The bias of neuron j
$v_{i,j,\ell}$	The ℓ-th LTD weight from neuron i to neuron j ($\ell \in [1, L]$)
$u_{i,j,k}$	The k-th LTP weight from neuron i to neuron j ($k \in [1, K]$)

(b) Learnable parameters

Notation	Definition
$x_j^{[t]}$	The value of neuron j at time t
$\gamma_{j,\ell}^{[t]}$	The ℓ-th neural eligibility trace of neuron j at time t
$\alpha_{i,j,k}^{[t]}$	The k-th synaptic eligibility trace from neuron i to neuron j at time t

(c) Variables

Table S1. The parameters and variables of a DyBM. (a) The structural parameters are fixed and are not updated when the DyBM is trained or is generating a sequence. (b) The learnable parameters are updated when the DyBM is trained but not when it is generating a sequence. (c) The variables are updated when the DyBM is trained or is generating a sequence.

Neuron		
Pre-synaptic	Post-synaptic	Delay
1	1	6
1	2	6
1	3	4
1	4	4
1	5	4
1	6	5
1	7	8
2	1	1
2	2	4
2	3	2
2	4	8
2	5	6
2	6	1
2	7	8
3	1	3
3	2	6
3	3	8
3	4	4
3	5	6
3	6	5
3	7	1
4	1	4
4	2	1
4	3	6
4	4	5
4	5	2
4	6	8
4	7	8
5	1	9
5	2	4
5	3	5
5	4	3
5	5	7
5	6	2
5	7	8
6	1	8
6	2	1
6	3	5
6	4	2
6	5	5
6	6	4
6	7	5
7	1	1
7	2	8
7	3	1
7	4	8
7	5	6
7	6	1
7	7	8

Table S2. The delay from a pre-synaptic neuron to a post-synaptic neuron. The delay is sampled independently from the uniform integer distribution with support $[1,9]$. The delay is fixed throughout the experiments.
